# Telepharmacy and Quality of Medication Use in Rural Areas, 2013–2019

**DOI:** 10.5888/pcd17.200012

**Published:** 2020-09-03

**Authors:** Shweta Pathak, Mitchell Haynes, Dima M. Qato, Benjamin Y. Urick

**Affiliations:** 1 University of North Carolina Eshelman School of Pharmacy, Chapel Hill, North Carolina; 2 University of Illinois College of Pharmacy, Chicago, Illinois

## Abstract

**Introduction:**

Pharmacy closures in rural areas is an increasingly common problem. Closures disrupt medication access and decrease adherence to prescription medications. Telepharmacy is a potential solution to this problem; however, research on the relationship between telepharmacy and the quality of medication use is scarce. Our study sought to address this gap by comparing the quality of telepharmacies serving rural areas and traditional pharmacies that support them.

**Methods:**

We obtained dispensing data for the first 18 months of operation from 3 telepharmacies and 3 traditional pharmacies located in the upper Midwest. We evaluated adherence for noninsulin diabetes medications, renin-angiotensin system antagonists, and statins, as well as inappropriate use of high-risk medications in older adults and statin use in persons with diabetes. All metrics were calculated using Medicare Part D specifications. We estimated the differences between telepharmacies serving rural areas and traditional pharmacies using generalized linear regression. We adjusted our models for potential sociodemographic and clinical confounders.

**Results:**

A total of 2,832 patients contributed 4,402 observations to the quality measures. After covariate adjustment, we observed no significant differences between telepharmacies and traditional pharmacies for noninsulin diabetes medications, renin-angiotensin system antagonists, statins, and high-risk medications. However, statin use in persons with diabetes was higher in telepharmacies than traditional pharmacies.

**Conclusion:**

We found that the quality of medication use at telepharmacies that serve rural areas was no worse than at traditional pharmacies. For communities considering the adoption of telepharmacy, results indicate that telepharmacies provide a suitable solution for expanding medication access and that using telepharmacy would not negatively affect the quality of medication use.

**MEDSCAPE CME**

In support of improving patient care, this activity has been planned and implemented by Medscape, LLC and *Preventing Chronic Disease*. Medscape, LLC is jointly accredited by the Accreditation Council for Continuing Medical Education (ACCME), the Accreditation Council for Pharmacy Education (ACPE), and the American Nurses Credentialing Center (ANCC), to provide continuing education for the healthcare team.

Medscape, LLC designates this Journal-based CME activity for a maximum of 1.00 AMA PRA Category 1 Credit(s)™. Physicians should claim only the credit commensurate with the extent of their participation in the activity.

Successful completion of this CME activity, which includes participation in the evaluation component, enables the participant to earn up to 1.0 MOC points in the American Board of Internal Medicine’s (ABIM) Maintenance of Certification (MOC) program. Participants will earn MOC points equivalent to the amount of CME credits claimed for the activity. It is the CME activity provider’s responsibility to submit participant completion information to ACCME for the purpose of granting ABIM MOC credit.

**Release date: September 3, 2020; Expiration date: September 3, 2021**

**Learning Objectives**

Upon completion of this activity, participants will be able to:

1. Distinguish remote services typically offered by telepharmacies
2. Compare characteristics of patients using traditional pharmacies and telepharmacies
3. Analyze medication adherence data in traditional pharmacies and telepharmacies
4. Evaluate outcomes improved in telepharmacies vs traditional pharmacies

**EDITOR**

Robin Sloan

Editor, Preventing Chronic Disease

Disclosure: Robin Sloan has disclosed no relevant financial relationships.

**CME AUTHOR**

Charles P. Vega, MD

Health Sciences Clinical Professor of Family Medicine

University of California, Irvine, School of Medicine

Irvine, California

Disclosure: Charles P. Vega, MD, has disclosed the following relevant financial relationships:

Served as an advisor or consultant for: Johnson & Johnson Pharmaceutical Research & Development, LLC; GlaxoSmithKline

Served as a speaker or a member of a speakers bureau for: Genentech, GlaxoSmithKline

**AUTHORS**

Shweta Pathak, MPH, PhD

University of North Carolina Eshelman School of Pharmacy

Chapel Hill, North Carolina

Disclosure: Shweta Pathak, MPH, PhD, has disclosed the following relevant financial relationships:

Other: Received funding from Cardinal Health

Mitchell Haynes, PharmD

University of North Carolina Eshelman School of Pharmacy

Chapel Hill, North Carolina

Disclosure: Mitchell Haynes, PharmD, has disclosed no relevant financial relationships.

Dima M. Qato, PharmD, PhD, MPH

University of Illinois College of Pharmacy

Chicago, Illinois

Disclosure: Dima M. Qato, PharmD, PhD, MPH, has disclosed the following relevant financial relationships:

Other: Received funding from Cardinal Health

Benjamin Y. Urick, PharmD, PhD

University of North Carolina Eshelman School of Pharmacy

Chapel Hill, North Carolina

Disclosure: Benjamin Y. Urick, PharmD, PhD, has disclosed the following relevant financial relationships:

Received grants for clinical research from: Cardinal Health, which owns the telepharmacy software used by pharmacies in this project.Kline

**Summary**

**What is already known on this topic?**

Pharmacy closures disrupt medication access and decrease patient adherence to prescription medications. Telepharmacy is a potential solution to this problem; however, research on the relationship between telepharmacy and adherence, as well as other aspects of the quality of medication use, is limited.

**What is added by this report?**

In rural areas, the quality of medication use at telepharmacies is no worse than at traditional pharmacies.

**What are the implications for public health practice?**

Our study informs public health officials and policy makers who are considering telepharmacy as an option for pharmacy support services in communities with limited medication access.

## Introduction

Across the United States, rural populations are decreasing and growing older (1). As a result, local businesses close in many small rural towns, and pharmacies that dispense medications to older adults are at risk of closing (2). In 2018, 16% of rural independent pharmacies had closed during the previous 16 years (3). Community pharmacies dispense 90% of medications in the United States (4), and pharmacy closures create disruptions in medication access that negatively affect medication adherence (5). Decreasing adherence rates lead to greater disease progression and create a substantial financial burden on the health care system (6).

A potential solution for maintaining medication access in rural communities is telepharmacy, which is the provision of patient care by pharmacists through the use of telecommunication or other technologies (7). In the community setting, telepharmacy most often replaces a physical check of patient adherence by a pharmacist with a remote check by a pharmacist. Filling prescriptions by a pharmacy technician also occurs under remote supervision. Additionally, patient counseling services are delivered by telephone or by video connection, as needed (8).

Although regulatory restrictions on telepharmacy have eased in recent years, as of 2016, less than half of all US states had rules or legislation authorizing telepharmacy practice (9). The safety of telepharmacy services has been explored to some extent (10,11), but the effects of telepharmacy on the quality of medication use is largely unknown. Limiting physical access to a pharmacist might negatively influence the quality of medication use, and this uncertainty has created barriers for the implementation of regulations that make telepharmacy licensure possible (12). The primary objective of our study was to evaluate the relationship between telepharmacy services in rural areas and the quality of medication use.

## Methods

Our cross-sectional study used retrospective data from the dispensing records of 3 pairs of telepharmacies and the traditional pharmacies that supported them. Telepharmacies were located in smaller rural communities and served a more rural population than the traditional pharmacies. The participating pharmacies are part of a commercial chain located in the upper Midwest region of the United States. Data were obtained from 15 to 18 months for each telepharmacy‒traditional pharmacy pair, starting with the opening date of the telepharmacy. An uptake period of 3 months was allowed for the establishment of operations, and the subsequent 12-month observation period was used for quality measurement. Each telepharmacy–traditional pair had different evaluation periods that were based on the opening date of telepharmacy services at each telepharmacy site. The date ranges for telepharmacy‒traditional pharmacy pairs were 1) April 1, 2013, to October 31, 2014; 2) May 1, 2015, to November 30, 2016; and 3) October 2, 2017, to January 11, 2019. The University of North Carolina at Chapel Hill’s Institutional Review Board approved the study.

We examined more than 150,000 dispensing records for 10,923 patients, of which, 8,786 patients met our overall population eligibility of adults aged 18 or older. Our primary exposure variable was the use of either telepharmacy or traditional pharmacy for medication management. Patient attribution to telepharmacy or traditional pharmacy was determined separately for each quality measure, according to the site where the patient filled at least 50% of their measure-eligible medications. Outcomes were assessed for 5 quality measures from 2 domains of quality of medication use: medication adherence and inappropriate medication use. Patients were eligible for inclusion in our sample if they met the inclusion criteria for any 1 of the 5 quality measures.

### Medication adherence

Medication adherence was evaluated for 3 common classes of medications: 1) noninsulin diabetes medications (NIDMs), 2) renin-angiotensin system antagonists (RASAs), and 3) statins. Each medication class is included in Medicare Part D Star Rating measures and Part D measure specifications (13), developed by the Pharmacy Quality Alliance (14) and endorsed by the National Quality Forum (15). Proportion of days covered (PDC), which is the preferred method to measure adherence (14), was used to assess patient adherence to these drug therapies. The PDC method assesses the percentage of patients covered by prescription claims for the same drug or for another drug in the same therapeutic class within a given period. Measure specifications for NIDM, RASA, and statin adherence require a denominator of patients aged 18 or older with at least 2 fills in the specified medication classes during the measurement year. Patients in the denominator with a PDC at 80% or higher (conventional cut-off) across the classes of medications were considered adherent to a given class of medication. A binary indicator of adherence was created for every patient who met measure specifications in the 12-month post-uptake window of their pharmacy.

### Inappropriate medication use

Inappropriate medication use was assessed using measures that are also part of Medicare Star Ratings. These measures were 1) use of high-risk medications (HRM) in the elderly and 2) statin use in persons with diabetes (SUPD). HRM eligibility, by definition, applies only to people aged 65 or older. The HRM measure includes all patients aged 65 or older as eligible for the measure denominator. Eligible patients who received 2 or more prescription fills for the same HRM class during the measurement period were included in the numerator. For the SUPD measure, denominator-eligible patients were aged 40 to 75 years with at least 2 diabetes medication fills during the measurement period. Patients in the denominator who received a statin medication fill during the measurement period were included in the numerator.

Like medication adherence measures, a binary indicator of inappropriate medication use was created for each patient who met the measure specification criteria within the 12-month post-uptake window of their pharmacy. For the SUPD measure, we found that all denominator-eligible patients in the telepharmacy Pair 1 site met the numerator specifications for this measure, making the lack of variation impossible to accurately assess the differential effect of pharmacy type on the outcome.

### Covariates

Covariates of patient age, sex, patient location (rural or urban), payer (Medicaid or other), patient risk indicator (low, moderate and high), and telepharmacy-traditional pair indicators were used to control for variations in observations on the basis of patient demographic and clinical factors. Because dispensing data for the same patient can appear across different points in time for different quality measures, the first fill date for eligible patients within each measure was used to calculate patient age. A Medicaid and non-Medicaid payer indicator was developed as a proxy for patient socioeconomic status. Patients were flagged as Medicaid payers if they had a prescription with Medicaid as the primary or secondary biller. Rural and urban classifications were made by linking county classification of rurality (16) to patient zip codes through a county-zip code crosswalk (17). The original classification scheme by the US Department of Agriculture’s Economic Research Service has 6 rural categories and 3 urban categories (16), which were combined into a binary indicator. Most urban patient zip codes were from counties of a population size of 250,000 or less, although rural patients were from a population size of 2,500 to 19,999. A pharmacy pair indicator was used to absorb any additional geographic or practice-related variation not accounted for by other covariates in the study.

Finally, an indicator of relative patient risk was derived by calculating medication counts for eligible patients within each quality measure. The medication count was determined by the count of distinct therapeutic classes of dispensed medications and was used to categorize prescription burden of patients into relative categories of low, moderate, and high risk based on the tercile of the distribution of the medication counts for each quality measure (18). This risk indicator was a proxy for disease severity in the covariate-adjusted models. A sensitivity analysis, using an alternative risk indicator for prescription burden categories of low polypharmacy (0–4), polypharmacy (5–9), and hyperpolypharmacy (≥9) using conventional polypharmacy cut-offs (19), was also performed.

### Statistical analysis

All variables were summarized by using counts and percentages for categorical variables and means, standard deviation, and interquartile ranges for continuous variables. Patient population characteristics were compared within telepharmacies and traditional pharmacies by using χ^2^ tests of proportions for categorical variables and Student *t* tests for continuous variables.

The effect of observations clustered within pharmacies on estimates was accounted for by using pharmacy as a repeated measure in generalized estimating equations (GEE), an extension of a generalized linear model. Additionally, binomial distributions with logit links were used to model all outcomes. This approach accounted for within-pharmacy heteroscedasticity to produce population-averaged estimates of binary outcomes. Unadjusted GEE models with only the pharmacy indicator and covariate-adjusted models were assessed for all 5 measures. Beta coefficients derived from unadjusted and adjusted models were converted to odds ratios for ease of interpretation. Additionally, least square-means for adjusted models and 95% confidence intervals for all models were estimated. All analyses were conducted using SAS version 9.4 (SAS Institute Inc).

## Results

Our final data set consisted of 2,832 patients who met eligibility criteria for at least 1 of our 5 measures. These patients contributed 4,402 observations to quality measures. Tercile-based risk stratification of patients yielded varied medication count cutoffs for each measure. The cut-off between low and moderate risk was 6 for NIDM, 5 for RASA, 5 for statins, 2 for HRM, and 6 for SUPD. The cut-off between moderate and high risk was 10 for NIDM, 9 for RASA, 9 for statins, 6 for HRM, and 10 for SUPD. More than 20% (661/2832 = 23.3%) of patients in our study received services through telepharmacies. Of 2,832 patients, pharmacy Pair 1 contributed 43.4% (n = 1,230), pharmacy Pair 2 contributed 37.0% (n = 1,049) and pharmacy Pair 3 contributed 19.5% (n = 553). The proportion of patients who used telepharmacies was 12.0% (148 of 1,230) in pharmacy Pair 1, 30.4% (319 of 1,049) in pharmacy Pair 2, and 35.1% (194 of 553) in pharmacy Pair 3. We observed no significant differences between telepharmacies and traditional pharmacies in population characteristics, such as patient age, sex, or payer (Table 1). Telepharmacies, however, had a significantly higher proportion (χ^2^ statistic, 352.2; *P* < .001) of patients from rural residential areas (84.1%; 556 of 661) than traditional pharmacies (27.8%; 603 of 2,171). Conversely, we observed a significantly higher proportion (χ^2^ statistic, 12.8; *P* = .002) of patient risk among those using traditional pharmacies (25.6%; 555 of 2,171) than telepharmacies (21.5%; 142 of 661).

**Table 1:** Comparison of Overall Patient Characteristics by Pharmacy type to Evaluate Quality of Medication Use, 2013–2019

Characteristics	Traditional, No. (%)^a^ (n = 2,171)	Telepharmacy, No. (%)^a^ (n = 661)	*P* Value
**Patient sex**			
Female	1,100 (50.7)	336 (50.8)	.94^b^
Male	1,071 (49.3)	325 (49.2)	
**Age group, y**			
18–49	182 (8.4)	57 (8.6)	.10^b^
50–64	509 (23.4)	134 (20.3)	
65–74	750 (34.5)	261 (39.5)	
>74	730 (33.6)	209 (31.6)	
**Patient location**			
Urban	1,568 (72.2)	105 (15.9)	<.001^b^
Rural	603 (27.8)	556 (84.1)	
**Patient risk^d^**			
High	555 (25.6)	142 (21.5)	.002^b^
Moderate	816 (37.6)	225 (34.0)	
Low	800 (36.8)	294 (44.5)	
**Payer**			
Medicaid	66 (3.0)	28 (4.2)	.13^b^
Other	2,105 (97.0)	633 (95.8)	
**Patient age, mean (SD), y**	68.5 (13.1)	68.2 (12.9)	.63^c^
**No. of medications, mean (SD)^e^**	6.3 (4.5)	5.5 (4.2)	<.001^c^

a Percentages may not add to 100 because of rounding.

b Derived from χ^2^ test.

c Derived from Student *t* test.

d Tercile-based stratification of the medication counts for measure-eligible patients; varies for each quality measure.

e Number of medications calculated as the count of distinct classes of dispensed medications.

For the adherence measures and SUPD, we found more male than female patients, and a greater proportion of the population was younger than 65. However, for the HRM measure, we found more female than male patients. Similarly, a higher proportion of patients were from urban residential areas in all measures except SUPD, where the proportion of patients from rural residential areas (50.3%) was almost equal to patients from urban residential areas (50.3%). Prevalence of adherence was 73.2% (188 of 257) for NIDMs, 75.6% (731 of 967) for RASAs, and 73.0% (755 of 1034) for statins. The prevalence of HRM use was 8.3% (164 of 1985), and the use of statins among diabetes patients was 66.0% (105 of 159) (Table 2). Covariate adjustment affected all quality measures (Table 3). After covariate adjustment, we observed no significant difference in adherence between telepharmacies and traditional pharmacies for NIDMs, RASAs, statin medications, or HRM. Predicted margins from adjusted models indicate proportions of adherence and inappropriate use for variables in the models (Table 4). Patients with diabetes who used telepharmacies; however, had a significantly higher likelihood of statin use (*P* < .001) than those using traditional pharmacies. Except for SUPD (83% vs 75%), the differences in the predicted margins for telepharmacies and traditional pharmacies were not significant. Sensitivity analysis using polypharmacy cut-offs did not meaningfully change the results for any of our quality measures.

**Table 2:** Description of Patient (N = 2,832) Characteristics by Outcomes for Medication Adherence and Inappropriate Use^a^, 2013–2019

Characteristics	Adherence to Noninsulin Diabetes Medications (n = 257)	Adherence to Renin-Angiotensin System Antagonist (n = 967)	Adherence to Statins (n = 1,034)	Use of High-Risk Medications^b^ (n = 1,985)	Statin Use In Persons With Diabetes (n = 159)
**Patient sex**					
Female	125 (48.6)	436 (45.1)	482 (46.6)	1,104 (55.6)	73 (45.9)
Male	132 (51.4)	531 (54.9)	552 (53.4)	881 (44.4)	86 (54.1)
**Age, y**					
18–49	38 (14.8)	151 (15.6)	108 (10.4)	—	22 (13.8)
50–64	116 (45.1)	365 (37.7)	432 (41.8)	—	73 (45.9)
65–74	50 (19.5)	239 (24.7)	255 (24.7)	1,046 (52.7)	59 (37.1)
>74	53 (20.6)	212 (21.9)	239 (23.1)	939 (47.3)	5 (3.1)
**Patient location**					
Urban	168 (65.4)	603 (62.4)	698 (67.5)	1,127 (56.8)	79 (49.7)
Rural	89 (34.6)	364 (37.6)	336 (32.5)	858 (43.2)	80 (50.3)
**Patient risk^c^**					
Low	73 (28.4)	298 (30.8)	305 (29.5)	574 (28.9)	48 (30.2)
Moderate	93 (36.2)	341 (35.3)	378 (36.6)	711 (35.8)	54 (34.0)
High	91 (35.4)	328 (33.9)	351 (33.9)	700 (35.3)	57 (35.8)
**Payer**					
Other	234 (91.1)	916 (94.7)	977 (94.5)	1,970 (99.2)	144 (90.6)
Medicaid	23 (8.9)	51 (5.3)	57 (5.5)	15 (0.8)	15 (9.4)
**Pharmacy type**					
Traditional	202 (78.6)	753 (77.9)	852 (82.4)	1,510 (76.1)	114 (71.7)
Telepharmacy	55 (21.4)	214 (22.1)	182 (17.6)	475 (23.9)	45 (28.3)
**Pharmacy pairs**					
Pair 3	35 (13.6)	169 (17.5)	169 (16.3)	412 (20.8)	38 (23.9)
Pair 2	90 (35.0)	335 (34.6)	307 (29.7)	804 (40.5)	121 (76.1)
Pair 1	132 (51.4)	463 (47.9)	558 (54.0)	769 (38.7)	—
**Prevalence^d^ of Adherence or Inappropriate Use**	188 (73.2)	731 (75.6)	755 (73.0)	164 (8.3)	105 (66.0)
**Age, mean (SD) [IQR], y**	62.3 (14.1) [53–73]	63.4 (13.7) [54–73]	64.5 (12.8) [55–74]	75.2 (7.9) [69–81]	60.9 (9.6) [54–69]
**No. of medications, mean (SD) [IQR]^e^**	9.3 (4.1) [6–12]	8.2 (4.5) [5–11]	8.3 (4.5) [5–11]	5.6 (4.3) [2–8]	9.4 (4.4) [6–12]

Abbreviations: — , not applicable; IQR, interquartile range.

a All values are number (percentage) unless otherwise indicated.

b Use of high-risk medications applies only to patients aged 65 or older, as per measure specifications.

c Tercile-based stratification of the medication count for measure-eligible patients; varies for each quality measure.

d Prevalence defined as all observations that met numerator specifications for each quality measure.

e Number of medications calculated as the count of distinct therapeutic classes of dispensed medications.

**Table 3:** Unadjusted and Covariate-Adjusted Estimates of the Effect of Pharmacy type on Quality of Medication Use

Variables	Quality Measures				
	Adherence to Noninsulin Diabetes Medications	Adherence to Renin-Angiotensin System Antagonist Medications	Adherence to Statins	Use of High-Risk Medications^b^ (≥65 y)	Statin Use in Persons with Diabetes^c^
**Unadjusted model pharmacy type^a^**					
Traditional	1 [Reference]	1 [Reference]	1 [Reference]	1 [Reference]	1 [Reference]
Telepharmacy	0.6 (0.4–0.8) [.001]	1.1 (0.8–1.4) [.60]	1.0 (0.7–1.7) [.84]	0.9 (0.8–1.1) [.20]	0.9 (0.7–1.3) [.80]
**Covariate adjusted model pharmacy type^a^**					
Traditional	1 [Reference]	1 [Reference]	1 [Reference]	1 [Reference]	1 [Reference]
Telepharmacy	0.8 (0.5–1.3) [.42]	1.0 (0.9–1.2) [.70]	1.3 (0.8–2.1) [.30]	1.3 (1.0–1.8) [.06]	1.7 (1.3–2.0) [<.001]
**Patient sex**					
Male	1 [Reference]	1 [Reference]	1 [Reference]	1 [Reference]	1 [Reference]
Female	1.4 (0.9–2.1) [.15]	0.7 (0.6–1.0) [.02]	0.9 (0.8–1.1) [.02]	1.1 (0.8–1.5) [.71]	0.3 (0.2–0.5) [<.001]
**Age group**					
18–49	1 [Reference]	1 [Reference]	1 [Reference]	—	—
50–64	3.2 (1.5–7.2) [.004]	1.8 (1.3–2.5) [.001]	2.1 (1.7–2.4) [<.001]	—	—
65–74	6.9 (2.5–16.5) [<.001]	2.5 (1.8–3.3) [<.001]	2.6 (2.2–3.2) [<.001]	1 [Reference]	—
≥65	—	—	—	—	3.9 (2.2–7.2) [<.001]
<65	—	—	—	—	1 [Reference]
>74	2.3 (1.4–3.7) [<.001]	2.2 (1.6–3.2) [.001]	2.2 (1.6–2.9) [<.001]	0.8 (0.6–1.0) [.03]	—
**Patient location**					
Urban	1 [Reference]	1 [Reference]	1 [Reference]	1 [Reference]	1 [Reference]
Rural	0.4 (0.3–0.6) [<.001]	1.3 (1.1–1.6) [.005]	0.7 (0.5–0.9) [<.003]	0.9 (0.6–1.2) [.40]	0.7 (0.5–1.1) [.11]
**Patient risk^d^**					
Low	1 [Reference]	1 [Reference]	1 [Reference]	1 [Reference]	1 [Reference]
Moderate	0.8 (0.4–1.9) [.69]	1.1 (0.7–1.5).80	0.9 (0.6–1.3) [.50]	5.5 (2.9–10.4) [<.001]	1.2 (0.7–2.1) [.49]
High	1.3 (0.5–3.3) [.52]	0.9 (0.7–1.1) [.40]	1.3 (1.0–1.5) [.02]	19.7 (10.6–36.3) [<.001]	2.1 (1.6–2.8) [<.001]
**Payer**					
Medicaid	1 [Reference]	1 [Reference]	1 [Reference]	1 [Reference]	1 [Reference]
Other	3.9 (2.1–6.9) [<.001]	1.9 (1.1–3.1) [<.02]	2.1 (1.2–3.8) [.01]	1.0 (0.4–2.2) [.94]	0.4 (0.3–0.6) [<.001]
**Pharmacy pair**					
Pair 3	1 [Reference]	1 [Reference]	1 [Reference]	1 [Reference]	1 [Reference]
Pair 2	3.1 (1.7–5.6) [.001]	1.7 (1.5–1.9) [<.001]	1.3 (1.0–1.7) [.08]	1.1 (1.0–1.3) [.13]	0.8 (0.7–1.0) [.01]
Pair 1	1.6 (0.9–2.9) [.14]	1.3 (1.1–1.4) [<.001]	0.6 (0.5–0.7) [<.001]	1.3 (1.1–1.5) [.01]	—

Abbreviation: — , not applicable.

a All values are odds ratio (95% CI) and [*P* value].

b Use of high-risk medications applies only to patients aged 65 or older, as per measure specifications.

c Age groups combined for model development; no assessment for pharmacy Pair 1.

d Tercile-based stratification of the medication count for measure-eligible patients; varies for each quality measure.

**Table 4:** Predicted Margins From Adjusted Models of Medication Adherence and Inappropriate Use Using Least Square Means^a^

Characteristics	Quality Measures				
	Noninsulin Diabetes Medications Adherence	Renin-Angiotensin System Antagonist Adherence	Statin Adherence	Use of High-Risk Medications^b^	Statin Use in Persons with Diabetes^c^
**Patient sex**					
Male	0.54 (0.46–0.61)	0.73 (0.65–0.79)	0.68 (0.60–0.75)	0.05 (0.03–0.08)	0.69 (0.65–0.74)
Female	0.61 (0.54–0.86)	0.67 (0.61–0.72)	0.67 (0.58–0.74)	0.05 (0.03–0.08)	0.87 (0.81–0.91)
**Age group**					
18–49	0.33 (0.21–0.49)	0.57 (0.50–0.63)	0.53 (0.43–0.62)	—	—
50–64	0.62 (0.56–0.68)	0.70 (0.63–0.77)	0.70 (0.61–0.77)	—	—
<65	—	—	—	—	0.66 (0.63–0.69)
65–74	0.78 (0.68–0.85)	0.76 (0.71–0.81)	0.75 (0.67–0.81)	0.05 (0.03–0.09)	—
≥65	—	—	—	—	0.88 (0.82–0.93)
>74	0.54 (0.49–0.58)	0.74 (0.67–0.80)	0.71 (0.63–0.78)	0.04 (0.03–0.07)	—
**Patient location**					
Urban	0.67 (0.60–0.73)	0.67 (0.58–0.74)	0.72 (0.63–0.79	0.05 (0.03–0.09)	0.82 (0.78–0.85)
Rural	0.47 (0.42–0.52)	0.73 (0.68–0.77)	0.63 (0.54–0.71)	0.04 (0.03–0.08)	0.77 (0.69–0.83)
**Patient risk^d^**					
Low	0.56 (0.41–0.71)	0.70 (0.65–0.75)	0.67 (0.56–0.76)	0.01 (0.00–0.02)	0.74 (0.67–0.80)
Moderate	0.52 (0.42–0.62)	0.71 (0.62–0.79)	0.64 (0.56–0.72)	0.06 (0.04–0.08)	0.77 (0.72–0.82)
High	0.63 (0.50–0.75)	0.68 (0.61–0.74)	0.72 (0.64–0.78)	0.17 (0.12–0.25)	0.85 (0.78–0.91)
**Payer**					
Non-Medicaid	0.73 (0.68–0.77)	0.76 (0.75–0.77)	0.75 (0.72–0.78)	0.05 (0.04–0.06)	0.72 (0.70–0.74)
Medicaid	0.41 (0.30–0.52)	0.63 (0.50–0.74)	0.59 (0.43–0.73)	0.05 (0.02–0.12)	0.85 (0.79–0.90)
**Pharmacy pair**					
Pair 3	0.44 (0.34–0.55)	0.64 (0.58–0.70)	0.70 (0.64–0.75)	0.04 (0.03–0.07)	0.81 (0.75–0.85)
Pair 2	0.71 (0.65–0.76)	0.75 (0.69–0.80)	0.75 (0.65–0.82)	0.05 (0.03–0.08)	0.78 (0.74–0.81)
Pair 1	0.56 (0.48–0.63)	0.69 (0.64–0.74)	0.57 (0.47–0.66)	0.05 (0.03–0.09)	—
**Pharmacy type**					
Traditional	0.60 (0.52–0.67)	0.69 (0.65–0.73)	0.65 (0.59–0.70)	0.04 (0.03–0.07)	0.75 (0.69–0.80)
Telepharmacy	0.55 (0.47–0.63)	0.70 (0.63–0.77)	0.70 (0.58–0.80)	0.06 (0.03–0.10)	0.83 (0.79–0.87)

Abbreviations: — , not applicable.

a All values are predicted margin (95% CI).

b Use of high-risk medications applies only to patients aged 65 or older, as per measure specifications.

c Age groups combined for model development; no assessment for Pharmacy Pair 1.

d Tercile-based stratification of the medication count for measure-eligible patients; varies for each quality measure.

## Discussion

This is the first study to evaluate differences in the quality of medication use between telepharmacies in rural areas and traditional pharmacies by using a broad set of standardized measures. We found that the quality of telepharmacies, as assessed by medication adherence and appropriateness, was no worse than in the traditional pharmacies that supported them. Substantial demographic and clinical differences, however, were observed in the populations served by the 2 pharmacy types. Telepharmacy patients were more likely to reside in rural areas and had a lower medication count. When accounting for these potential confounders, no significant differences were observed between telepharmacy and traditional pharmacies, except for the SUPD measure, on which telepharmacies scored higher. Additional data are needed to confirm that the lack of significance for the HRM measure was not a result of type 2 error.

Our findings on medication adherence support findings from a previous study (20), which found no difference in adherence rates among patients at an urban telepharmacy and those at a retail chain pharmacy. Unlike that study, our study assessed adherence to medications by using standardized measure specifications and examined additional measures of quality, such as inappropriate use. Moreover, our study used data from multiple pairs of telepharmacies in rural areas and traditional pharmacies, increasing our sample size and allowing us to use stronger evaluation methods for assessing our outcomes.

Coupled with safety data from previous studies (10,11), our study can inform boards of pharmacy about the positive relationship between telepharmacy practice and the quality of medication use. Our study might be useful as boards consider this alternative practice model to support their public mission of expanding medication access and improving population health in underserved communities in rural areas. Additionally, for community pharmacy owners and health care institutions considering new telepharmacy operations, our research suggests that new telepharmacies are likely to perform similarly to existing pharmacies that will support them. Establishment of telepharmacies, therefore, might not necessarily place organizations at an additional risk for performance-related penalties, which have become common among third-party payers in the United States (21).

Our study can also inform public health officials, researchers, and policy makers considering telepharmacy as an alternative to increase medication access in communities with poor access to medications. A common term for these communities is pharmacy deserts, and pharmacy deserts are prevalent in both rural and urban areas (22,23). Urban telepharmacies might have similar relative qualities to rural telepharmacies; however, boards of pharmacy, public health leaders, and policy makers should carefully consider regulations that limit the geographic scope of telepharmacies until a better understanding of the implications on medication access and quality of telepharmacies in urban areas is obtained.

Our study had several limitations, primarily as a result of the use of dispensing records for assessment of outcomes and the small number of pharmacies. Dispensing data provide limited information on sociodemographic and clinical factors that can affect the quality of medication use. We addressed this limitation to the extent possible by creating indicators for patient rurality, Medicaid-status and patient risk. Additionally, dispensing data do not capture the complete spectrum of pharmacies visited by patients. It is unlikely, however, that the use of outside pharmacies varied systematically by pharmacy type, and therefore any bias would be balanced across cohorts.

Differences in community pharmacy practice in rural and urban areas (24) might have influenced our findings of telepharmacy and traditional pharmacy outcomes, but we were unable to disentangle those differences in our study. Finally, because telepharmacy practice can differ across states (9), our findings are only generalizable to similar pharmacies serving similar populations. Additional study is needed to evaluate the relative quality of telepharmacies in urban areas and other demographically diverse settings.

Our study indicates that the quality of medication use at telepharmacies serving rural areas is similar to the quality provided through traditional pharmacies. Our findings can be used to inform public health policy makers on the suitability of telepharmacy as one solution for improving medication access and facilitating population health in rural pharmacy deserts. Moreover, our results support telepharmacy deregulation and imply that, for institutions participating in alternative payment models, contracting with telepharmacies to dispense medications should not negatively affect patient health or affect quality. Future studies should consider evaluating differences in medication quality for telepharmacies using other outcomes, such as glycosylated hemoglobin, and in other settings, such as urban telepharmacies.

## Post-Test Information

To obtain credit, you should first read the journal article. After reading the article, you should be able to answer the following, related, multiple-choice questions. To complete the questions (with a minimum 75% passing score) and earn continuing medical education (CME) credit, please go to http://www.medscape.org/journal/pcd. Credit cannot be obtained for tests completed on paper, although you may use the worksheet below to keep a record of your answers.

You must be a registered user on http://www.medscape.org. If you are not registered on http://www.medscape.org, please click on the “Register” link on the right hand side of the website.

Only one answer is correct for each question. Once you successfully answer all post-test questions, you will be able to view and/or print your certificate. For questions regarding this activity, contact the accredited provider, CME@medscape.net. For technical assistance, contact CME@medscape.net. American Medical Association’s Physician’s Recognition Award (AMA PRA) credits are accepted in the US as evidence of participation in CME activities. For further information on this award, please go to https://www.ama-assn.org. The AMA has determined that physicians not licensed in the US who participate in this CME activity are eligible for AMA PRA Category 1 Credits™. Through agreements that the AMA has made with agencies in some countries, AMA PRA credit may be acceptable as evidence of participation in CME activities. If you are not licensed in the US, please complete the questions online, print the AMA PRA CME credit certificate, and present it to your national medical association for review.

## Post-Test Questions

### Study Title: Telepharmacy and Quality of Medication Use in Rural Areas, 2013–2019

#### CME Questions

1. Which of the following services in telepharmacy is routinely performed remotely?
  - Supervision of medication fills by pharmacy technicians only
  - Patient counseling only
  - Supervision of medication fills and patient counseling only
  - Supervision of medication fills, patient counseling, and physical checks of patient adherence
2. Which one of the following patient characteristics was most different in comparing traditional pharmacies and telepharmacies in the current study?
  - Higher proportion of high-risk patients in traditional pharmacies
  - Higher proportion of women in traditional pharmacies
  - Higher proportion of patients with Medicaid in telepharmacies
  - Higher proportion of older patients in traditional pharmacies
3. Which one of the following statements regarding medication adherence rates in comparing traditional pharmacies and telepharmacies in the current study is most accurate?
  - Adherence was significantly higher in traditional pharmacies
  - Adherence was significantly higher in telepharmacies
  - Adherence was similar in traditional pharmacies and telepharmacies
  - Adherence was significantly higher in telepharmacies only in rural areas
4. Which one of the following outcomes was superior in comparing telepharmacies with traditional pharmacies in the current study?
  - Statin use in diabetes
  - Patient satisfaction
  - Use of potentially harmful drugs
  - Medication errors at filling
